# Temporal interference stimulation of peripheral nerves induces functionally diverse limb movements revealed by automated pose estimation and unsupervised behavioral analysis

**DOI:** 10.1186/s12984-025-01825-3

**Published:** 2025-12-29

**Authors:** Joshua Philippe Olorocisimo, Sudip Nag, Hengjia Zhang, Songyu Yang, Matvii Prytula, Serena Liu, Mustafa Kanchwala, Yinghe Sun, Jose Zariffa, Roman Genov

**Affiliations:** 1 Department of Electrical and Computer Engineering, University of Toronto, Toronto, ON Canada; 2 Institute of Biomedical Engineering, University of Toronto, Toronto, ON Canada; 3 KITE Research Institute, Toronto Rehabilitation Institute – University Health Network, Toronto, ON Canada; 4 Rehabilitation Sciences Institute, University of Toronto, Toronto, ON Canada

## Abstract

Peripheral nerve stimulation can help restore limb movement after paralysis and enable advanced rehabilitation technologies; however, current extraneural interfaces are typically limited by low fascicle selectivity and laborious functional evaluation. This study has developed an extraneural peripheral nerve interface with high fascicle selectivity, and an AI-facilitated video analysis pipeline for assessing limb movement during neuromodulation. This was achieved by deploying temporal interference stimulation (TIS) in a high-density nerve cuff electrode and by using machine learning algorithms for automated pose estimation and unsupervised behavioral analysis to evaluate movement selectivity and diversity. Using this unbiased semi-automated analysis revealed that TIS elicited more selective motor responses than standard biphasic stimulation, as evidenced by the formation of 1.75 times more distinct movement clusters and behavioral syllables. Furthermore, logit link beta regression modeling showed that TIS had a significantly higher positive effect on movement selectivity (β = 2.75, *p* < 0.005) compared to biphasic stimulation. Our statistical and machine learning-based analysis provides a computational and objective pipeline for quantifying complex motor outcomes in neuromodulation research. The results suggest that extraneural TIS can be used to generate individually targeted and functionally diverse limb movement patterns and offers a promising approach for neurorehabilitation applications, including restoring movement to individuals living with spinal cord injury.

**Supplementary Information:**

The online version contains supplementary material available at 10.1186/s12984-025-01825-3.

## Introduction

Peripheral nerve stimulation is a technique used to treat conditions of neurological disorders such as chronic pain, epilepsy, and spinal cord injury [1–6]. For instance, it was demonstrated that neuromodulation of the trigeminal and occipital nerves alleviated neuropathic pain and migraine, respectively; while vagus nerve stimulation was effective in treating epilepsy [7–10]. Peripheral nerve stimulation can also restore sensorimotor functions by reinstating muscle movement in spinal cord injury patients or providing sensory feedback in amputees [11–14]. Importantly, over 86,000 people suffer from spinal cord injury with an estimated cost of up to 3 million dollars of economic burden in Canada alone, and over 15 million people affected worldwide [15–17]. Thus, there is an urgent need to develop and advance peripheral nerve stimulation techniques and devices.

Current peripheral nerve interfaces are generally grouped into 3 categories, which from least to most invasive are: extraneural, intraneural, and regenerative [1, 18, 19]. As the invasiveness of these devices increases, so does the resolution or selectivity of stimulation. This trade-off occurs because increased control of the fascicles within the peripheral nerve requires more invasive interfaces and procedures that position the device closer to the fascicles, but risks more tissue damage and surgical complexity. Although there is currently a trade-off between invasiveness and selectivity, the optimal interface should theoretically have low invasiveness and high selectivity.

The common method for peripheral nerve stimulation is through the use of biphasic pulses which are charge-balanced, rectangular, electrical stimulation waveforms consisting of a negative phase and a positive phase [20]. Biphasic stimulation alternates between negative and positive current of equal charge to induce neuronal activation without any excess charge build up. However, this stimulation waveform affects axons that are physically closer to the electrodes more strongly than areas that are far away [21]. Therefore, targeting inner fascicles without activating outer fascicles is difficult to achieve using biphasic stimulation, and thus reduces its functional selectivity.

A recent method of neuronal stimulation is temporal interference stimulation (TIS) [22]. TIS employs high-frequency electric fields to selectively stimulate deep neural structures without activating the surrounding tissue [23]. Although the exact biophysical mechanisms are debated [24–26], this approach has the potential to induce specific, targeted movements in patients with compromised motor function by selectively stimulating different fascicles within a peripheral nerve [27–29]. These advances provide an avenue for improving the trade-off between invasiveness and stimulation selectivity in a peripheral nerve interface, by using extraneural electrodes, which are less invasive than their intraneural counterparts, and combining them with TIS to increase selectivity. Using TIS, previous studies have induced movement and selective muscle activation in the rodent hindlimb [25, 30]. However, only a limited number of movements were characterized, and the stimulation selectivity was not compared to the gold-standard biphasic stimulation. Therefore, the movement diversity and selectivity of TIS has not been fully elucidated, and there is a need to compare the types and quality of induced motion relative to biphasic stimulation.

In order to facilitate comparisons of TIS and biphasic stimulation selectivity, the aim of the current study is to develop a novel analysis pipeline, applying an unbiased and automated method of studying movement and behavior. We used recent advances in pose-tracking [31, 32] and unsupervised behavioral classification and segmentation [33]. We then illustrate the capabilities of this analysis pipeline by using it to test whether TIS would allow for more specific and diverse movements compared to biphasic stimulation when targeting the rat sciatic nerve using a 64-channel nerve cuff electrode.

## Methods

### Device fabrication and set-up

**Fig. 1:**
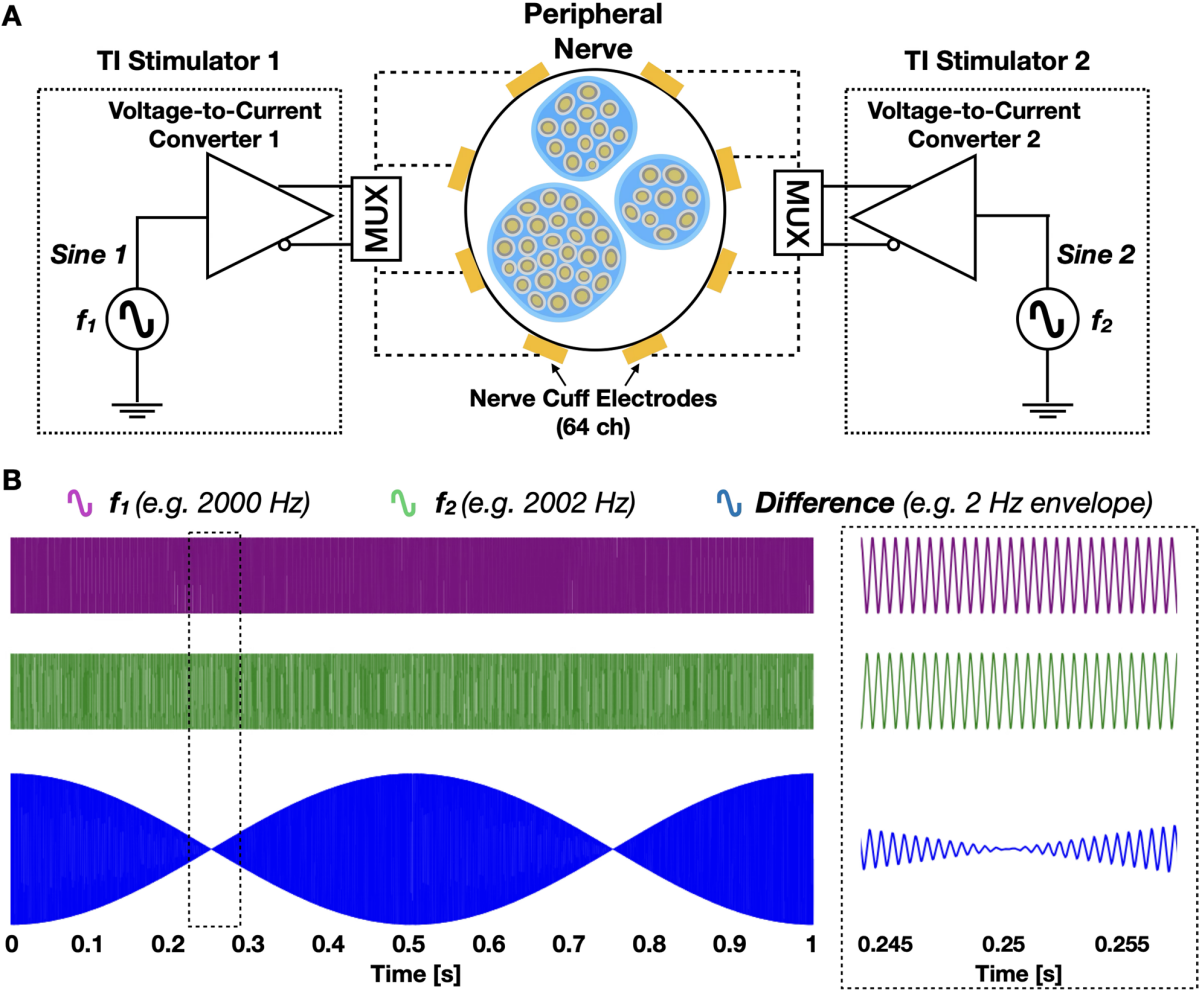
Schematic diagram of the temporal interference stimulation (TIS) set-up and sample waveforms. **A** Overview of the electronic components of the nerve cuff stimulator interfacing with a peripheral nerve and its fascicles. **B** Sample TIS waveform where high frequency f_1_ and f_2_ are shown on the first and second row, and the resulting interference waveform is shown at the bottom. The black dotted box shows a zoomed-in inset.

To perform temporal interference stimulation (TIS), two synchronized sine-wave stimulators were designed (Fig. 1A). The stimulators consisted of a sine-wave generator and a voltage-to-current converter as the main blocks. The sine waves for TIS were generated by using a dual-channel function generator (Model 33522B, Keysight, USA), whereas, the voltage-to-current converters were designed by using commercially-available integrated circuit chips (OPA4132, Texas Instruments Inc., USA). As shown in Fig. 1A, the system operated in current mode, where the input control signal was a voltage waveform (100–300 mV) and the output delivered to the electrodes was a proportional current waveform (100–300 µA).Thus, although the TIS dual-sine wave function generator was defined in mVpp, the converters ensured current-controlled delivery through the electrodes. The stimulators were operated at slightly different frequencies, f_1_ and f_2_ (e.g., 2 kHz and 2.002 kHz) as depicted in Fig. 1B. The high frequency waves interfere and interact within the nerve bundle, forming a low frequency envelope, f_1_ − f_2_ (e.g., 2 Hz). The high frequency waves are thought to be filtered out by the neuronal membrane, where only the low frequency envelope frequency induces the stimulation effect; however, as will be discussed, recent studies have shown other possible biophysical mechanisms — especially in peripheral nerves [23, 25, 26]. This method is referred to as temporal interference stimulation (TIS).

**Fig. 2:**
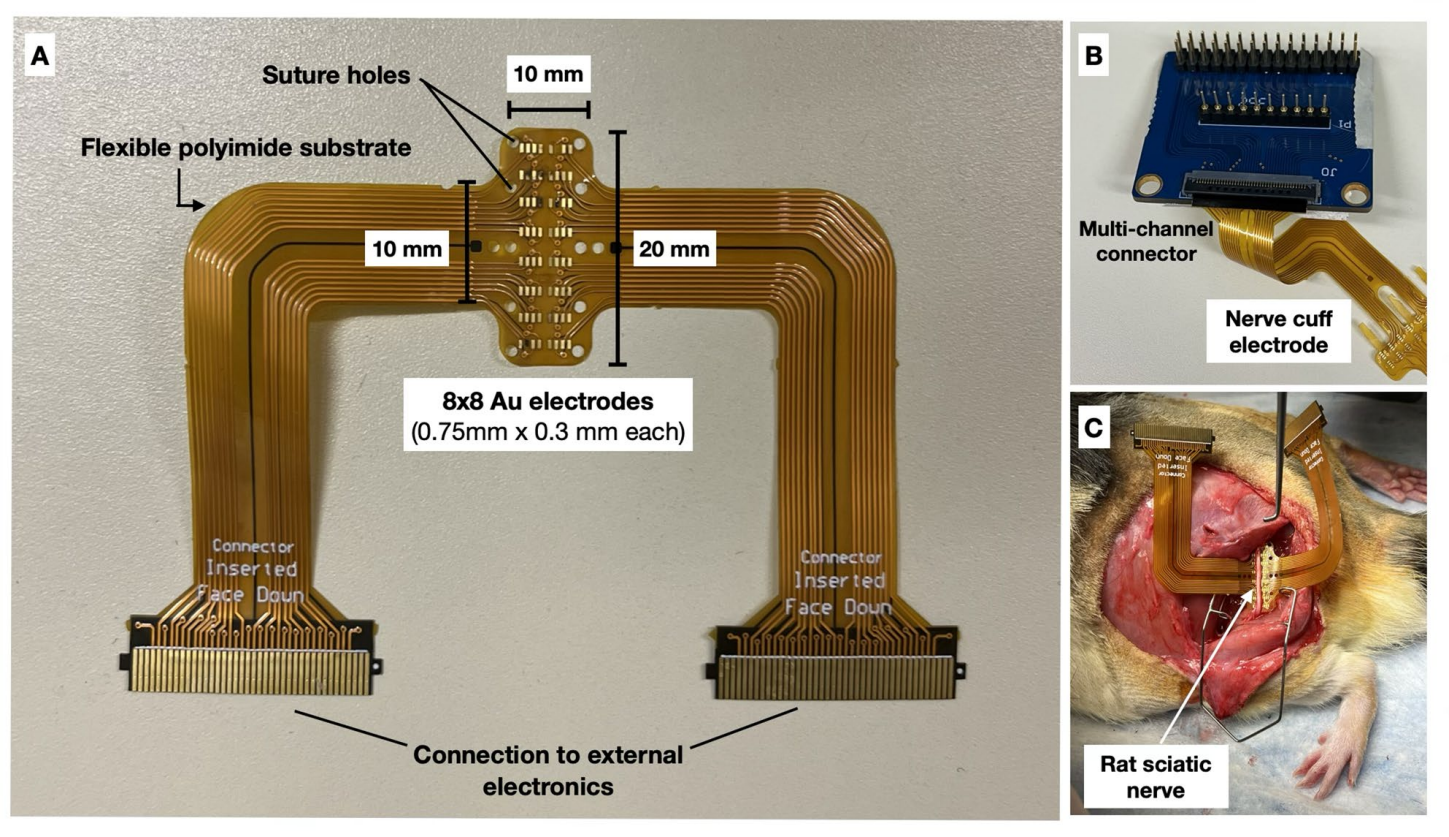
Extraneural nerve cuff electrode for temporal interference stimulation of the sciatic nerve. **A** Photograph of the implantable nerve cuff electrode and its dimensions. **B** Photo of the multiplexer allowing for channel switching. **C** Photo of the device implantation in the rat sciatic nerve.

The outputs of the stimulators were connected to a flexible extraneural nerve cuff with electrodes made of gold as the interface material with the epineurium of the nerve. This was designed using Altium Designer and sent for fabrication (Safe PCB, USA) with a thickness of 0.07 mm and electrode pitch of 0.4 mm. The distance between electrodes were 0.4 mm column-wise and 1.2 mm row-wise, with a mid-column spacing of 0.6 mm (Fig. 2A). The electrodes and stimulator were bridged by a multi-channel connector for multiplexing (Fig. 2B). This connector allowed for selecting the contact location of the stimulation across the 64-channel nerve cuff.

Furthermore, before implantation, the stimulator was tested with emulated electrode-electrolyte-tissue impedance and in vitro saline solution set-up using a resistor-load test and oscilloscope recordings. Charge balancing accuracy was measured over approximately 10,000 cycles of stimulation prior to moving onto in vivo experiments. Average charge injection error was found to be less than 1 nC, as confirmed with 1 kΩ + 10 kΩ//100 nC impedance, averaged over 1000 trials. Then, after in vivo experiments, explanted electrodes further confirmed no electrode dissolution nor tissue damage, which confirmed reliable charge balancing accuracy. The set-up was designed and tested for up to 10 kHz signals.

### Animal surgery and device implantation

For in vivo animal testing, 12 male Long-Evans retired breeder rats were used. The rats were anesthetized initially with 5% isofluorane induction and then kept at 2.5% for the rest of the surgery. A toe pinch was performed to check the animal’s pain reflex, and if no reflex was found, the surgery proceeded. Furthermore, blood oxygen level and heart rate was monitored throughout the procedure to ensure the animal was healthy and anesthetized. The fur was shaved and the skin disinfected with povidone-iodine. The groove between the biceps femoris and gluteus maximus was located for the incision site and cut using surgical scissors. The muscle bodies were separated using retractors and fascia cleared out until the sciatic nerve was revealed. Connective tissue surrounding the nerve was removed for optimal electrode-nerve contact, and the nerve cuff electrode was gently inserted below the nerve (Fig. 2C). Finally, the cuff was wrapped around the nerve by tightening it through the suture holes. Procedures were approved by the Animal Care Committee of the University Health Network (#6793).

### Electrode configurations and stimulation parameters

**Fig. 3:**
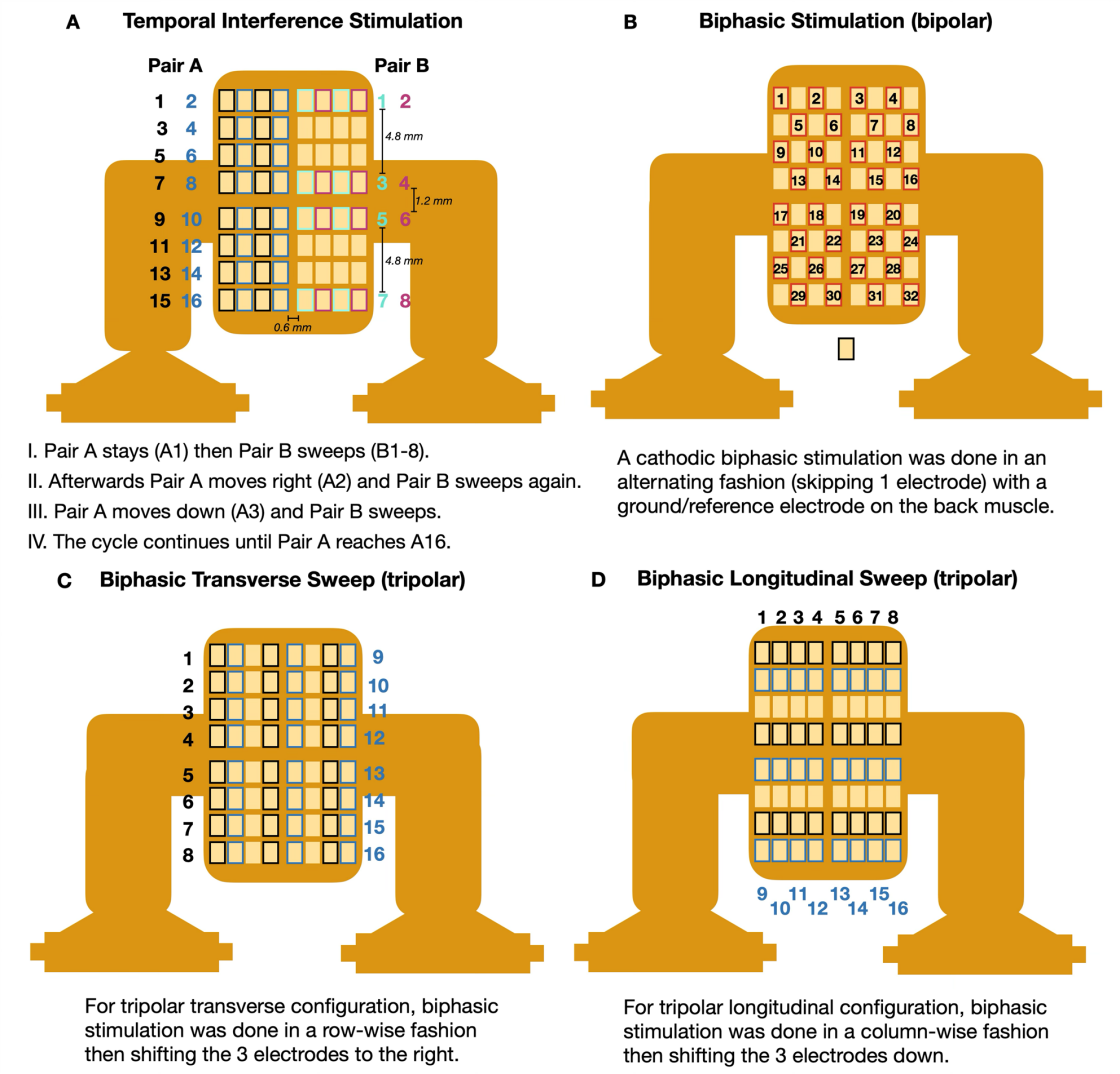
Electrode configurations for temporal interference and biphasic neurostimulation. **A** For TIS, 4 electrodes were required since two pairs of electrodes need to produce 2 different high frequency sine waves for interference. **B**–**D** For biphasic stimulation, 2–3 electrodes were used for stimulation, as allowed by the experimental set-up, following previous studies (Dali et al., 2019). The electrode configuration switching were eventually automated for a high-throughput sweep that reduced long wait times, thereby preventing overexposure and nerve damage.

To enable dynamic and automated control of electrode configurations for neuromodulation experiments, we developed a custom multiplexer switching system that integrates firmware-based control, Python automation, and structured data logging. The firmware was implemented using Code Composer Studio, where C code was written and uploaded onto two microcontrollers that controls the ON/OFF switching of the multiplexer pins each time a UART packet is received. The Python program sends control signals that represent the electrode pin configuration through UART to both microcontrollers. The system iterates through all possible configurations while automatically excluding faulty channels. Each change in configuration is recorded in a structured .txt file with timestamps. This automation enabled multiple stimulation configurations which can be tested sequentially within a single experiment. Stimulation generally lasted 5 s and then 5 s rest was given before moving onto the next configuration. The electrode configurations tested are outlined in Fig. 3 and Supplementary Fig. 1.

The electrode configurations are as follows: TIS required 4 channels (4-ch) since two pairs (Pair A and Pair B) of electrodes were needed to produce two interfering current waves. The pairs were 1 electrode apart. Pair A started in the first row and first column then Pair B swept across the rows, skipping some rows to avoid over-stimulation. Then after the Pair B sweep was done, Pair A moved right and the Pair B sweep was done again. Afterwards, Pair A moved down to the next row and first column, where it stayed while Pair B swept through the channels. Then the cycle continues. The configurations were swept across the nerve cuff with a total of 128 unique configurations for automated TIS with additional configurations tested manually for a total of 165 configurations. Labeling follows 8(A-1) + B formula (e.g. Pair A = 2; Pair B = 3; label = 11).

Pair A stimulation was set to 2 kHz and Pair B to 2.001–2.002 kHz (Δf = 1–2 Hz) for producing distinct but smooth contractions within the range of previous studies [30]. The amplitude ranged from 100 mVpp to 300 mVpp depending on electrode impedance which was measured using electrical impedance spectroscopy (24.6 kΩ to 32 kΩ). Lower amplitudes were used if sufficient limb movement was observed and higher amplitudes were introduced if movement thresholds increased or if larger interference signals were required as measured by an oscilloscope. A DC offset of 625 mV–1.65 V was introduced solely to center the sinusoidal waveform within the compliance range of the voltage-to-current converters and prevent saturation or clipping. Due to AC coupling at the converter outputs, the electrode interface received only the alternating component of the signal, yielding a net DC output current of zero. Lastly, the duty cycle used for the stimulation was 5 s ON/5 s OFF to allow recovery and avoid muscle fatigue.

Biphasic channels were tested with either 2 or 3 channels, as inspired by previous studies [34]. For the bipolar (BP, 2-ch) set-up, the electrodes were tested in an alternating fashion, skipping one electrode each across the cuff. For the tripolar (3-ch) set-up, two sub-types were tested: transverse and longitudinal, wherein the operating electrodes are two electrodes apart. For tripolar transverse (TT), the sweep was done in a row-wise fashion, then after all the rows were done, the trio shifts to the right. For tripolar longitudinal (TL), the sweep was done in a column-wise fashion, then the trio shifts one row down. The automated biphasic stimulation sweep had 64 unique configurations with additional configurations tested manually for a total of 151 configurations.

Similar to TIS, biphasic stimulation amplitude was dependent on electrode-tissue impedance and movement threshold, and typically ranged from 100 µA to 200 µA with a constant-current configuration, while ensuring charge balance between phase 1 and phase 2. For each phase, the width was 150 µs and the interphase interval was 53 µs. A repetition frequency of 16 Hz to 30 Hz was used with a pulse count of 100 biphasic pulses per stimulation trial to reliably produce sustained movements, and a 5s OFF duration duty cycle was used to allow for recovery.

As illustrated in Fig. 3, the automated multiplexer-driven stimulator enabled a comprehensive, high-throughput exploration of both temporal interference and traditional biphasic neuromodulation across a 64-electrode cuff array. The calculated charge densities were 0.01875 mC/cm^2^/phase (Biphasic pulse, 200 µA peak, 150 µs/phase) and 0.0298 mC/cm² per half-carrier cycle (TIS, 300 µA peak, 2 kHz) which are well below the Shannon safety criteria [35]. Given this, the set-up allowed the researchers to systematically probe the parameter space governing selective nerve activation while maintaining charge-balanced and safe electrical stimulation. Automated channel switching further accelerated data collection, and minimized cumulative nerve exposure, thereby reducing the risk of overstimulation and tissue damage.

### Data recording and computational analysis

**Fig. 4:**
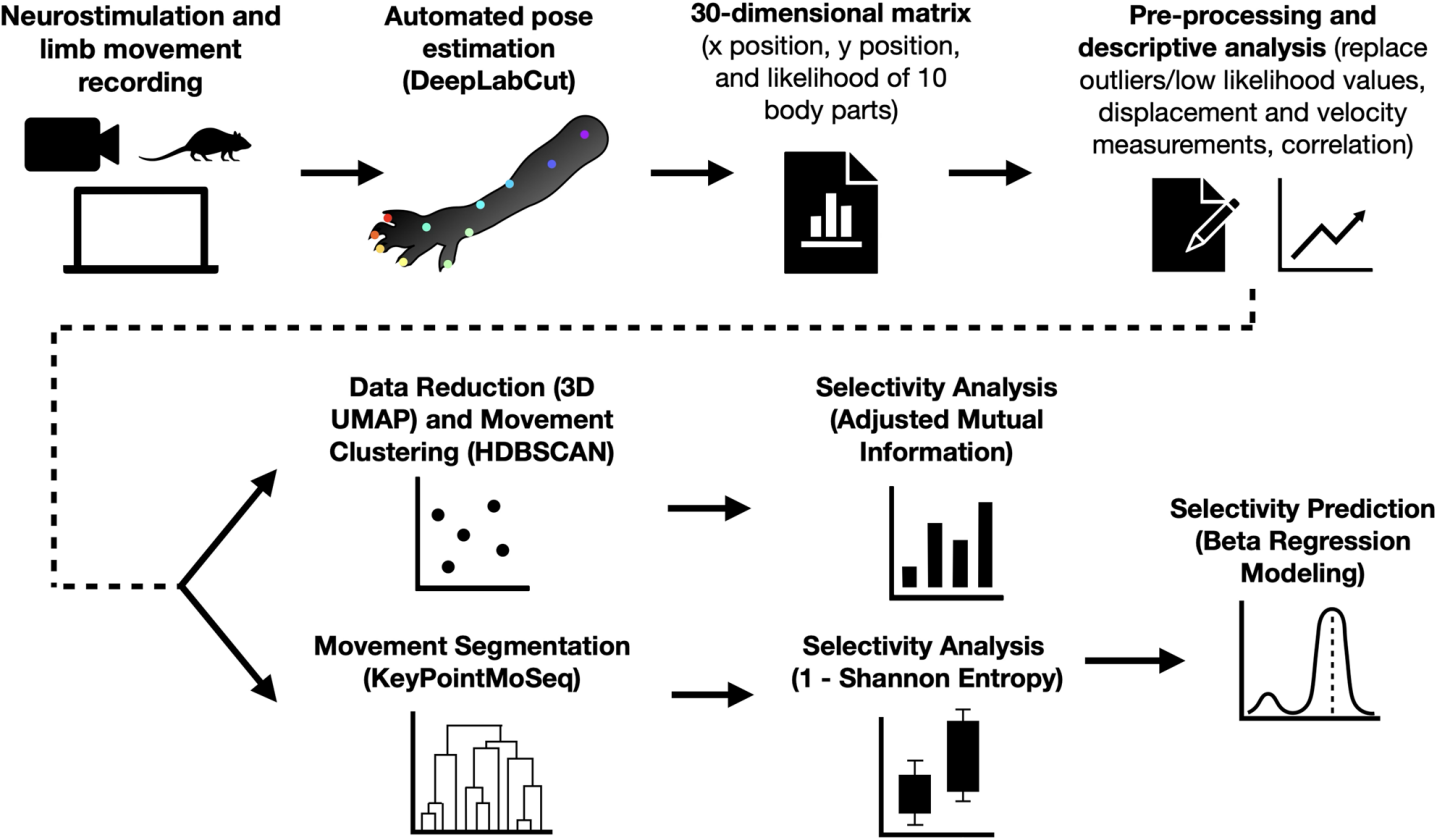
Overview of the analysis pipeline illustrating neurostimulation, recording, tracking, clustering, and modeling methodologies.

Figure 4 shows the overview of the analysis pipeline. For motion analysis, hindlimb movement was recorded using a camera at 60 fps (Elgato, Germany) from the top view. Then, specific body parts (called ‘keypoints’) were tracked by implementing the DeepLabCut (DLC) [31, 32] pipeline. Ten keypoints were labelled, namely: the hip, knee, ankle, heel, instep, and the five toes (toe 1 as the proximal/medial phalanx and toe 5 as the distal/lateral phalanx) [36]. Automated selection of representative frames for labeling was chosen using K-means clustering. Once frames were selected, they were manually labeled using DeepLabCut’s graphical interface. For consistency, defined anatomical markers such as joints were used to prevent manual misalignments that could introduce tracking artifacts. The annotated dataset was then used to train a ResNet-50 deep transfer learning model, leveraging GPU acceleration (NVIDIA GTX 1070) with the CUDA toolkit for faster convergence, and image augmentation (imgaug) for enhancing model performance. Training initially spanned 200,000 iterations, but additional iterations were performed as needed based on model performance, which was assessed via mean standard error (MSE) calculations and comparisons between predicted and manually labeled keypoints.

Once trained, the model was deployed for full-scale motion tracking, analyzing stimulation-induced movement trajectories across the whole video. The trained network processed unseen video frames, generating keypoint coordinates with confidence scores, which were exported into .csv files for quantitative analysis. To validate accuracy, the DeepLabCut plotting module was used to overlay predictions onto ground truth labels, and kinematic trajectory plots illustrated movement dynamics over time. A post-processing step was included to filter out low-confidence predictions and remove erroneous keypoint jumps. Additionally, skeletal overlays were added to output videos that visually connected keypoints from the hip to the toes and provided a clear representation of limb motion.

After data extraction and standardization, the Uniform Manifold Approximation and Projection (UMAP) algorithm was applied to reduce dimensionality while preserving both local and global structures. Then, we employed Hierarchical Density-Based Spatial Clustering of Applications with Noise (HDBSCAN) to identify movement patterns across experimental conditions. Hyperparameters were optimized through a combination of grid search and Bayesian optimization. Specifically, these were evaluated based on clustering performance metrics such as the silhouette score and adjusted mutual information, ensuring that the dimensionality reduction and clustering effectively preserved meaningful structure in the data.

For UMAP, the primary parameters tuned included n_neighbors, which controls the size of the local neighborhood; the min_dist parameter, responsible for determining how tightly points are distributed; and the n_components, which was set to 3 as initial experiments with 2D embeddings did not fully capture the complexity of rodent movement, whereas 3D projections provided a more comprehensive view of pose variation. Separate optimized parameter configurations were used for biphasic and TIS trials to accommodate differences in data distribution. In biphasic trials, UMAP was configured with n_neighbors = 50, min_dist = 0, and n_components = 3; furthermore, HDBSCAN was set with min_cluster_size = 30, min_samples = 5, and cluster_selection_epsilon = 1 to detect fine-grained movement clusters. For TIS trials, a larger neighborhood size (n_neighbors = 177) and more stringent clustering conditions (min_cluster_size = 128, min_samples = 300) were used due to increased complexity in movement responses. These optimized settings ensured robust identification of movement clusters, allowing the assessment of different stimulation paradigms.

We then implemented KeyPointMoSeq (KPMS) to segment movement patterns by fitting an Autoregressive Hidden Markov Model (AR-HMM) to our keypoint-based time-series data. First, we performed an AR-only fitting phase for 50 iterations, allowing the algorithm to learn a preliminary autoregressive structure of the keypoint trajectories. Next, we ran an additional 500 iterations for the full fitting phase, during which the model refined both the AR parameters and the segmentation of behavioral “syllables.” We set the latent dimension to 3, enabling a three-dimensional latent representation of the rodent’s movement. To regulate the AR fitting and segmentation, we adjusted the kappa hyperparameter, typically using a value of 1e9 for the AR-only stage and 1e8 for the full fitting, though these values varied depending on the number and quality of syllables generated. By iterating between AR parameter updates and segmentation adjustments, KPMS generated a robust, low-dimensional model of the rodent’s keypoint dynamics, facilitating downstream analysis of distinct behavioral motifs or syllables.

Lastly, selectivity metrics were calculated using the Adjusted Mutual Information (AMI) score for HDBSCAN clustering and the Shannon Entropy for KPMS segmentation, as will be discussed in the results. Electrode configurations that did not induce movement were removed in the selectivity analysis, which was determined using a threshold of the velocity (Supplementary Fig. 2). Then, selectivity was modeled by setting the stimulation type (TIS vs. Biphasic), electrode configuration, and subject IDs as predictors in a beta regression model. Calculations and statistics were done through Python and Jupyter notebook using numpy, pandas, statsmodels, scipy, sklearn, matplotlib, and seaborn packages, and results were tabulated and saved on Excel. Figure 4 outlines the entire analysis pipeline.

## Results

### Motion tracking and deeplabcut analysis

**Fig. 5:**
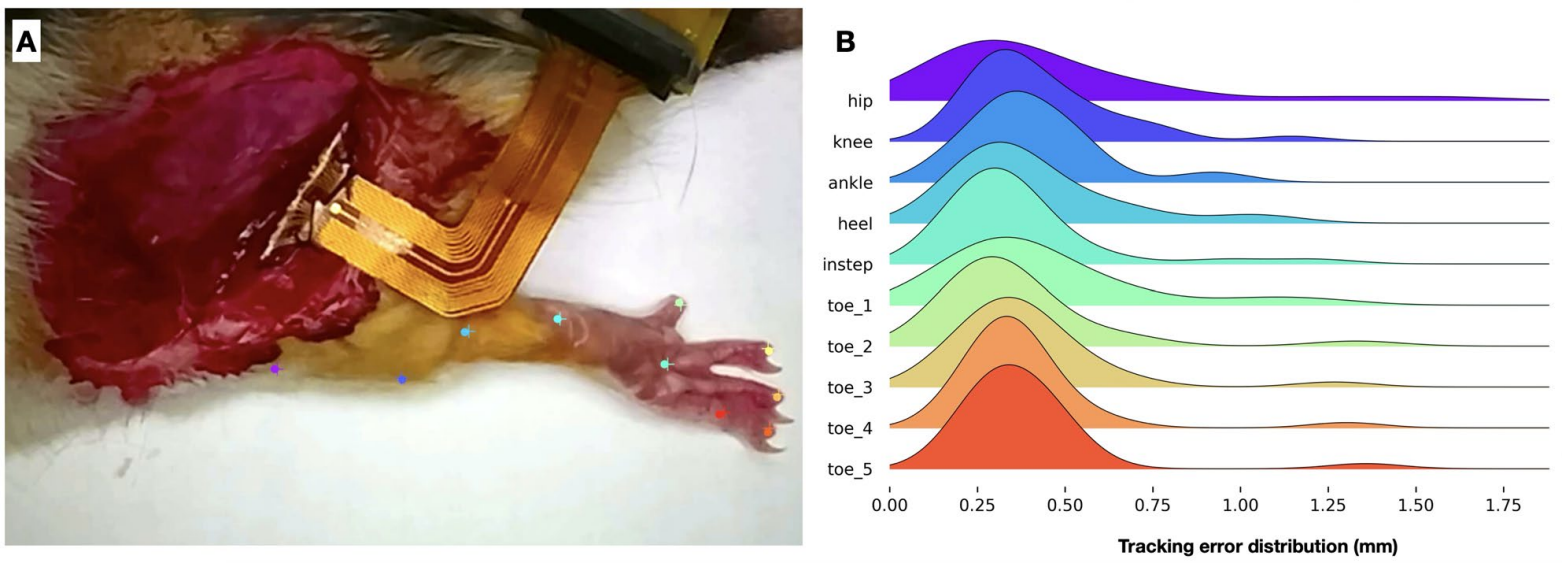
DeepLabCut (DLC) tracking accuracy. **A** Sample photograph of the DeepLabCut tracking results. Plus symbols represent the human-labeled points, and the dots represent the labels by DLC. **B** Probability density function of the tracking error across all testing trials. The median errors (mm) are as follows: hip = 0.314, knee = 0.353, ankle = 0.371, heel = 0.332, instep = 0.333, toe_1 = 0.370, toe_2 = 0.298, toe_3 = 0.341, toe_4 = 0.346, toe_5 = 0.352, overall = 0.340 mm.

An average of 120 frames were labelled per trial and the trained ResNet-50 neural network successfully tracked the rest of the ~ 60,0000 frames per trial. The DLC model accuracy was evaluated, and the median tracking error was found to be 0.34 mm across all 10 keypoints (Fig. 5).

Video tracking results were double-checked to see if model-generated labels matched human labels, and if mistakes were found, additional training iterations were added to ensure the model was performing well. This pipeline enabled a precise and high-throughput evaluation of stimulation-induced movement with human verification combined with automated electrode switching and deep-learning-based motion tracking for a scalable system to study peripheral nerve stimulation.

### Movement clustering analysis using UMAP-HDBSCAN

The computer vision model had an output of 30 dimensions across time (x position, y position, and likelihood of the 10 keypoints). Thus we removed values with low likelihoods (< 0.5), indicating low model confidence, and replaced them by interpolating the values across time using the median of the x, y position vectors for that body part. Then we flattened the x, y coordinates using the Euclidean distance formula in each keypoint, resulting in a 10-dimensional position matrix across time. Then we applied UMAP for non-linear data reduction of the 10 keypoints across time, for each animal separately, into 3 dimensions and performed clustering analysis.

**Fig. 6:**
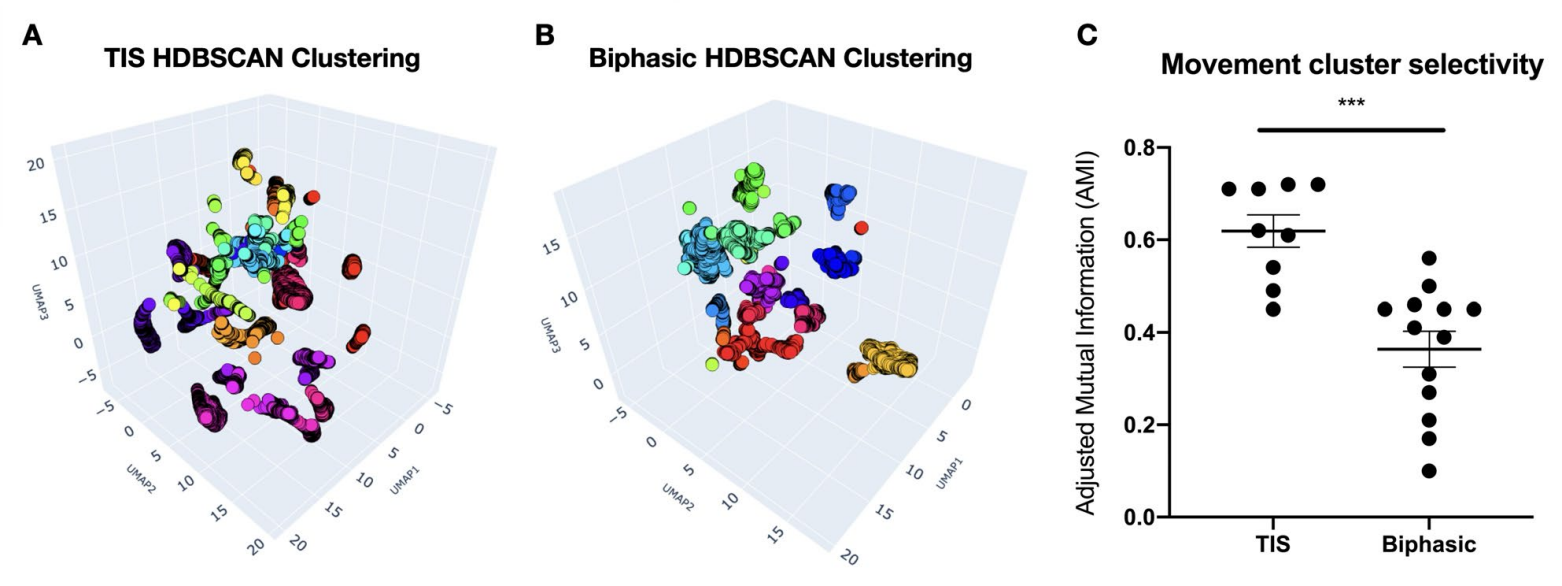
Movement clustering analysis showed higher selectivity (AMI) for TIS trials compared to Biphasic trials. **A**, **B** UMAP-HDBSCAN analysis shows the 3D-projected global structure of the motion data and their cluster assignments (colors). **C** AMI metric revealed TIS to be significantly more selective than Biphasic (*p* = 0.0002, Unpaired t-test). The examples shown in (A) and (B) (from different animals) correspond to the subjects closest to the mean of the group AMI distributions in (C) for TIS and Biphasic, respectively.

HDBSCAN clustering analysis separated movement clusters for each stimulation in an unsupervised manner (Fig. 6A-B). These clusters suggest unique motor outputs elicited by the stimulation modality from different electrode configurations and locations. Additionally, the actual electrode configuration and K-means clusters were also graphed (Supplementary Fig. 3); however HDBSCAN provided better clusters that matched the electrode configurations. To quantify and compare how well these movement clusters corresponded to the electrode configurations tested, the group AMI was calculated.

AMI is a metric that ranges from 0 (no alignment) to 1 (perfect alignment), and it measures the agreement between clustering results and predefined ground truth labels [37]. Thus, the movement clusters were compared to electrode configurations as ground truth labels. The AMI score from multiple subjects revealed that TIS produced movement clusters that better matched the tested configurations (mean AMI = 0.63) compared to biphasic stimulation (mean AMI = 0.36) (Fig. 6C). This indicated that the movement patterns elicited by TIS were more distinct and corresponded better to specific electrode configurations by 1.75 times more than biphasic stimulation.

**Fig. 7:**
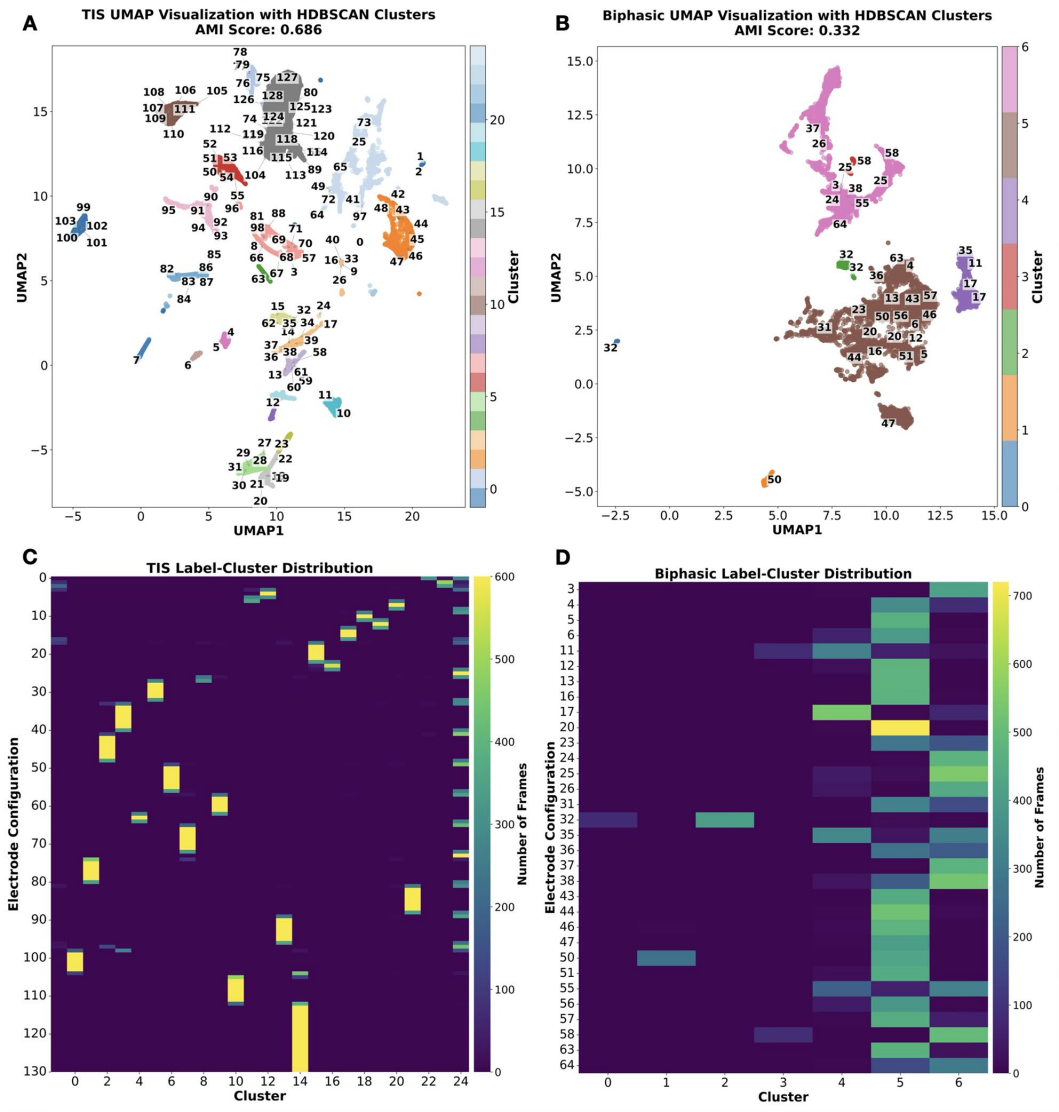
2D UMAP-HDBSCAN projection and electrode configuration distribution, for the same samples as those shown in Fig. 6. **A**, **B** Color bar represents cluster group labels after UMAP-HDBSCAN, while the numbers in the graph represent the actual electrode configuration labels described in Fig. 3. Consecutive numbers are in close physical proximity. **C**, **D** Heat map of the cluster-configuration assignments. TIS configurations (4-ch) formed distinct clusters, whereas biphasic configurations (2-ch) had similar cluster assignments, indicating less diverse and less selective movements in high-dimensional space.

To further illustrate the results, representative single-subject trials were graphed, and the clusters were tagged with their corresponding electrode configuration labels (Fig. 7A-B). It can be observed that TIS resulted in a higher number of clusters and AMI score, where electrode labels in close proximity formed distinct clusters separate from those with distant electrodes. In contrast, bipolar biphasic stimulation produced fewer clusters and a lower AMI score. Although the electrode labels were numerically and physically far apart, the induced movement still tended to cluster together (Fig. 7C-D). Even with shared UMAP-HDBSCAN parameters, the TIS sample still had a higher AMI score (Supplementary Fig. 4). This indicated that TIS achieved more distinct movements specific to the electrode location or configuration, while biphasic stimulation had less diverse and less selective movement clusters.

To assess whether this effect could be due to random noise, the position vectors were either (i) randomly shuffled across time (row-wise) or (ii) perturbed with Gaussian noise. Both procedures abolished the AMI difference, suggesting that the original TIS distribution represents a meaningful structure rather than stochastic noise. We also tested additional bipolar and tripolar configurations for biphasic stimulation compared to TIS in the same rat. The clustering and AMI scores of biphasic stimulation slightly improved, but TIS still displayed higher metrics (Supplementary Fig. 5). When calculating the selectivity score which will be discussed later, the biphasic tripolar configurations tended to be less selective than bipolar and TIS configurations (Supplementary Fig. 6). Therefore, only TIS displayed a consistent effect of increased selectivity throughout the analysis.

### Behavioral segmentation and selectivity analysis

Although clustering analysis revealed distinct movement clusters for TIS, this method lacked dependency on dynamics and temporal information. Therefore, to incorporate temporal dynamics, we utilized KPMS for further analysis of the behavioral data. KPMS employs an AR-HMM that models the transition between different behavioral states (syllables), allowing for the identification of distinct movement patterns over time.

**Fig. 8:**
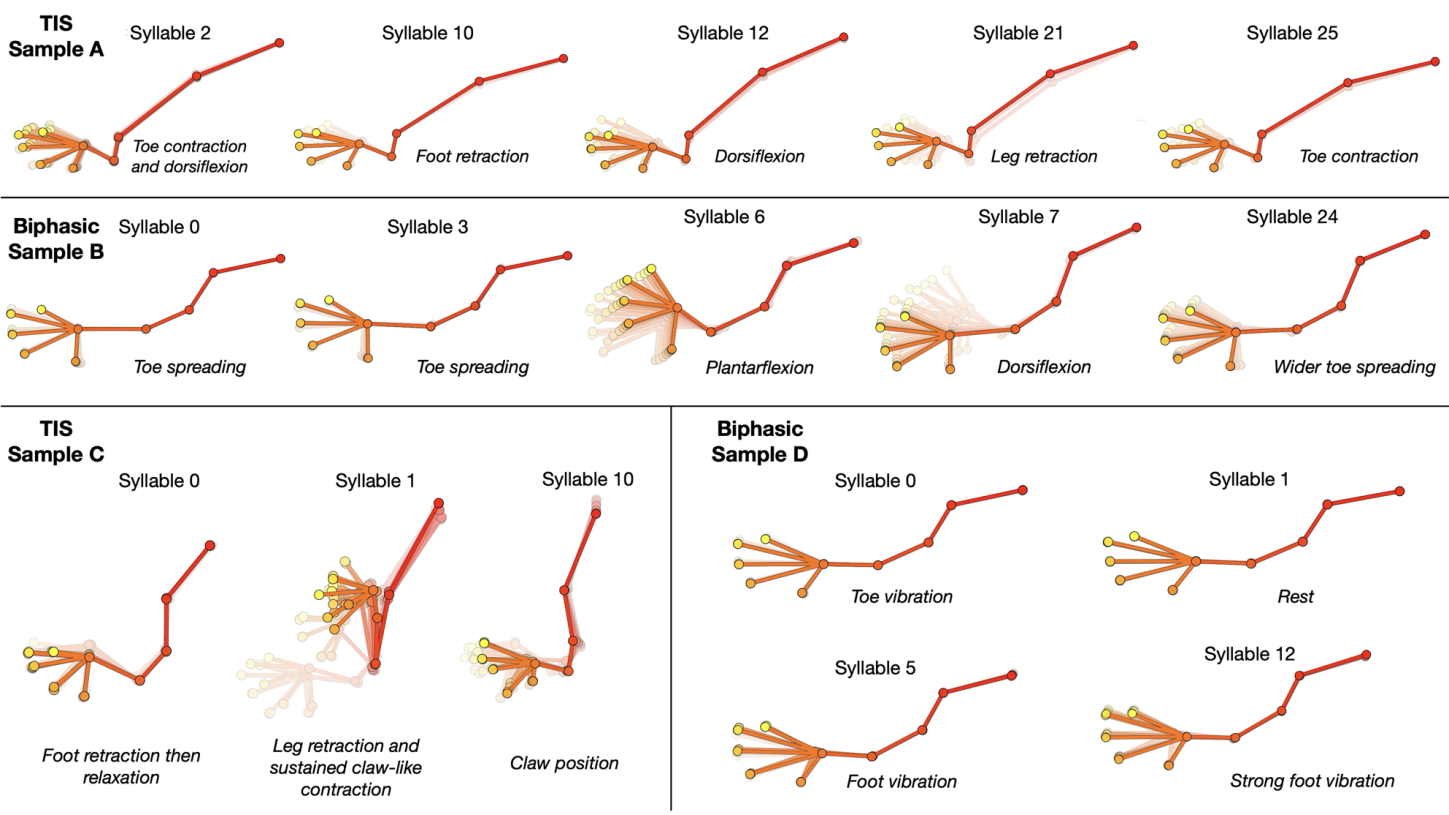
Behavioral syllables extracted after KeyPoint Moseq analysis. Syllables were identified in an unsupervised manner then were described manually post-analysis. When comparing examples with similar number of syllables identified, the behavioral syllables from TIS are more varied and complex.

**Fig. 9:**
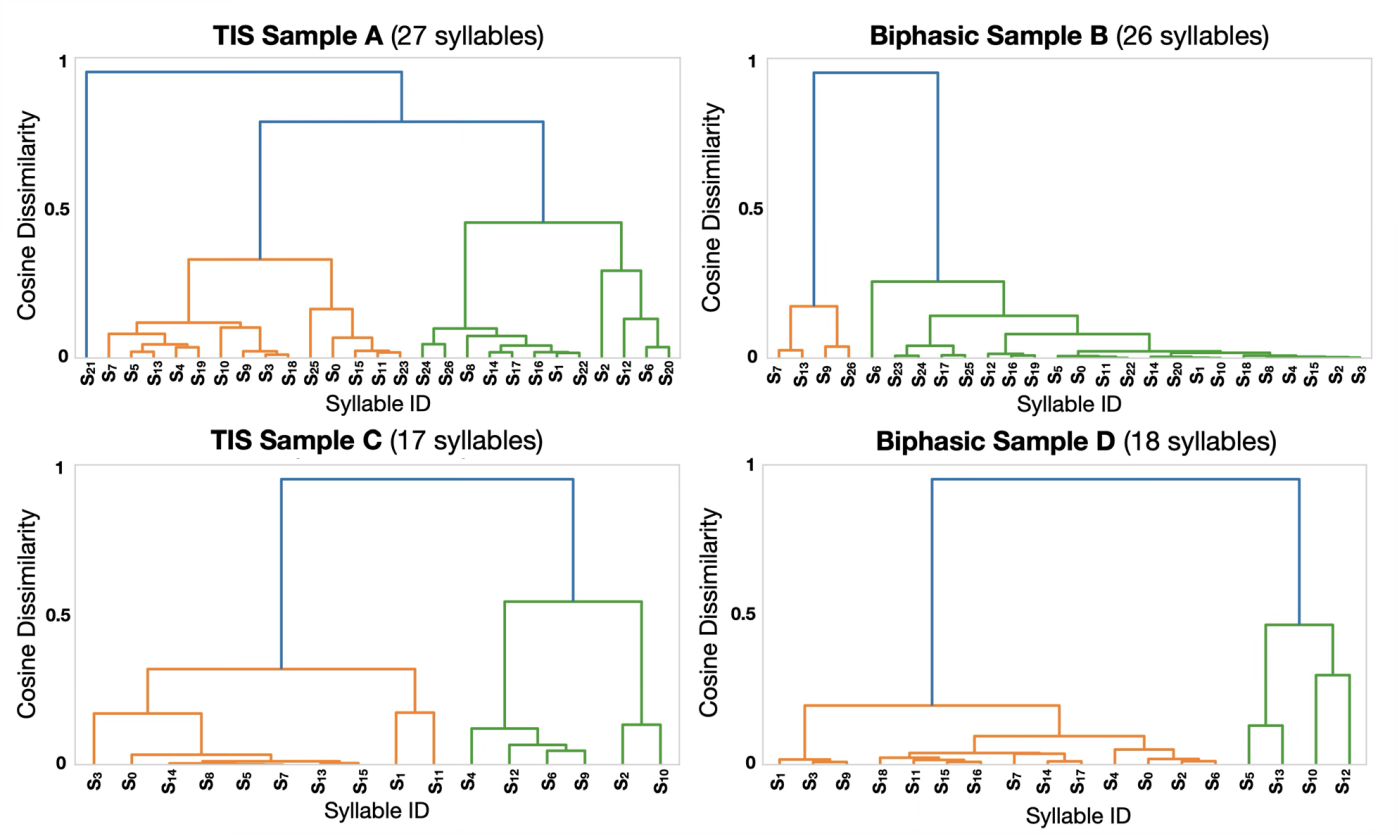
Dendrograms showing syllable similarity/dissimilarity using cosine metric. Taller divergences represent more distinct syllables.

Using a kappa (“stickiness”) hyperparameter of 10^8^, we were able to automatically extract different behavioral syllables in an unsupervised manner for TIS and biphasic stimulation trials (Fig. 8). These were then reviewed and labeled post-hoc manually by cross-referencing with the videos. Furthermore, the syllable similarities were measured using the cosine metric across the keypoint coordinate vectors and graphed in a dendrogram (Fig. 9). It can be seen that TIS trials resulted in a higher variety of movement and cosine dissimilarity compared to biphasic trials. In addition, KPMS was able to separate syllables of different speeds and intensities, as well as distinguish movement combinations from single motions.

Based on these results, TIS displayed more heterogenous and complex movements than biphasic stimulation. Even when comparing trials with a similar number of extracted syllables (Figs. 8 and 9 and A vs. B and C vs. D), we found that the TIS samples had higher dissimilarities between the syllables. On the other hand, biphasic samples had less distinct syllables from each other. Similar to the clustering results earlier, this indicated that TIS had a higher diversity of induced movements compared to biphasic stimulation.

Lastly, by using the extracted syllables, we analyzed if specific electrode configurations corresponded to specific syllable IDs. We used Shannon Entropy ($\:H$) to measure the uncertainty or randomness in the distribution of syllable IDs for each electrode configuration. A higher entropy value indicates a more varied distribution of syllable IDs, suggesting less selectivity for that electrode configuration. Conversely, a lower entropy value indicates a more specific and concentrated distribution of syllables for that electrode configuration, suggesting higher selectivity [33, 38, 39]. The formula for entropy ($\:H$) for a configuration $\:i$ is:

$$\:{H}_{i}=\:-{\sum\:}_{j}^{n}{p}_{ij}\mathrm{log}\left({p}_{ij}\right)$$

and we calculated the selectivity metric as the following:

$$\:{Selectivity\:Score}_{\:i}=1-\:\frac{{H}_{i}}{\mathrm{log}\left(N\right)}\:\:$$

where $\:{p}_{ij}$ is the proportion of occurrences of syllable $\:j$ within configuration $\:i$, and $\:n$ is the number of syllables for one configuration and $\:N$ is the total number of syllables for all electrode configurations in the trial.

**Fig. 10:**
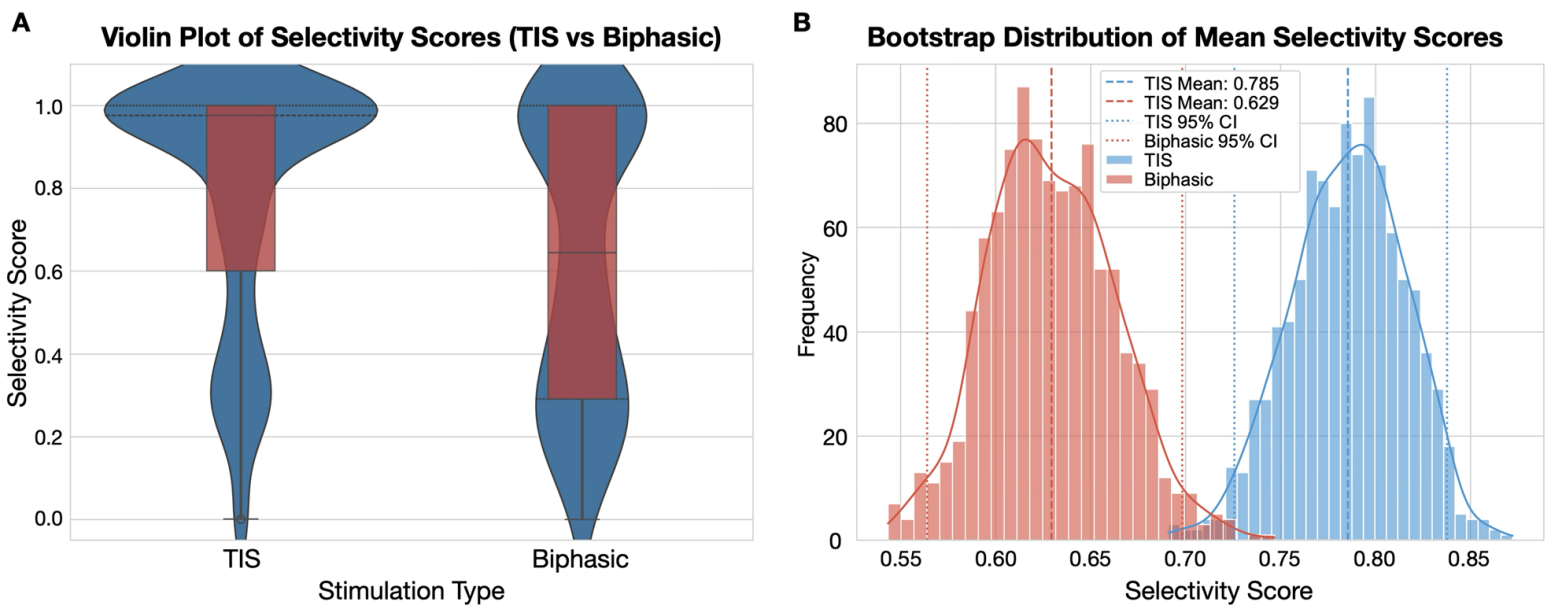
Selectivity score distribution of TIS and biphasic stimulation based on the specific correspondence of electrode configuration to the behavioral syllables. **A** Violin-box plot of the selectivity scores revealed TIS had a higher peak distribution than biphasic stimulation. **B** Bootstrapping revealed the mean TIS selectivity score was 0.7854, while the biphasic selectivity score was 0.6294, and the 95% confidence intervals were not overlapping.

Measurement of the selectivity score revealed TIS selectivity to be higher than biphasic stimulation trials (Fig. 10). A violin-box plot of the scores showed that a higher distribution of TIS scores were closer to 1, while biphasic scores had a larger variance with a notable distribution of scores below 0.4 (Fig. 10A). Due to the higher number of possible configurations for TIS (4 C 64 electrodes = 635376) compared to biphasic configurations (2 C 64 = 2016; 3 C 64 = 41664), we tested more TIS trials (*n* = 676) compared to biphasic (m = 164). To ensure fair comparison, we performed bootstrap resampling of the selectivity scores with 100 sample size and 1000 iterations. Bootstrap analysis revealed separate distributions for TIS and biphasic scores where the 95% confidence intervals did not overlap. The average of the bootstrap sample means was 0.79 for TIS and 0.63 for biphasic stimulation, respectively.

**Fig. 11:**
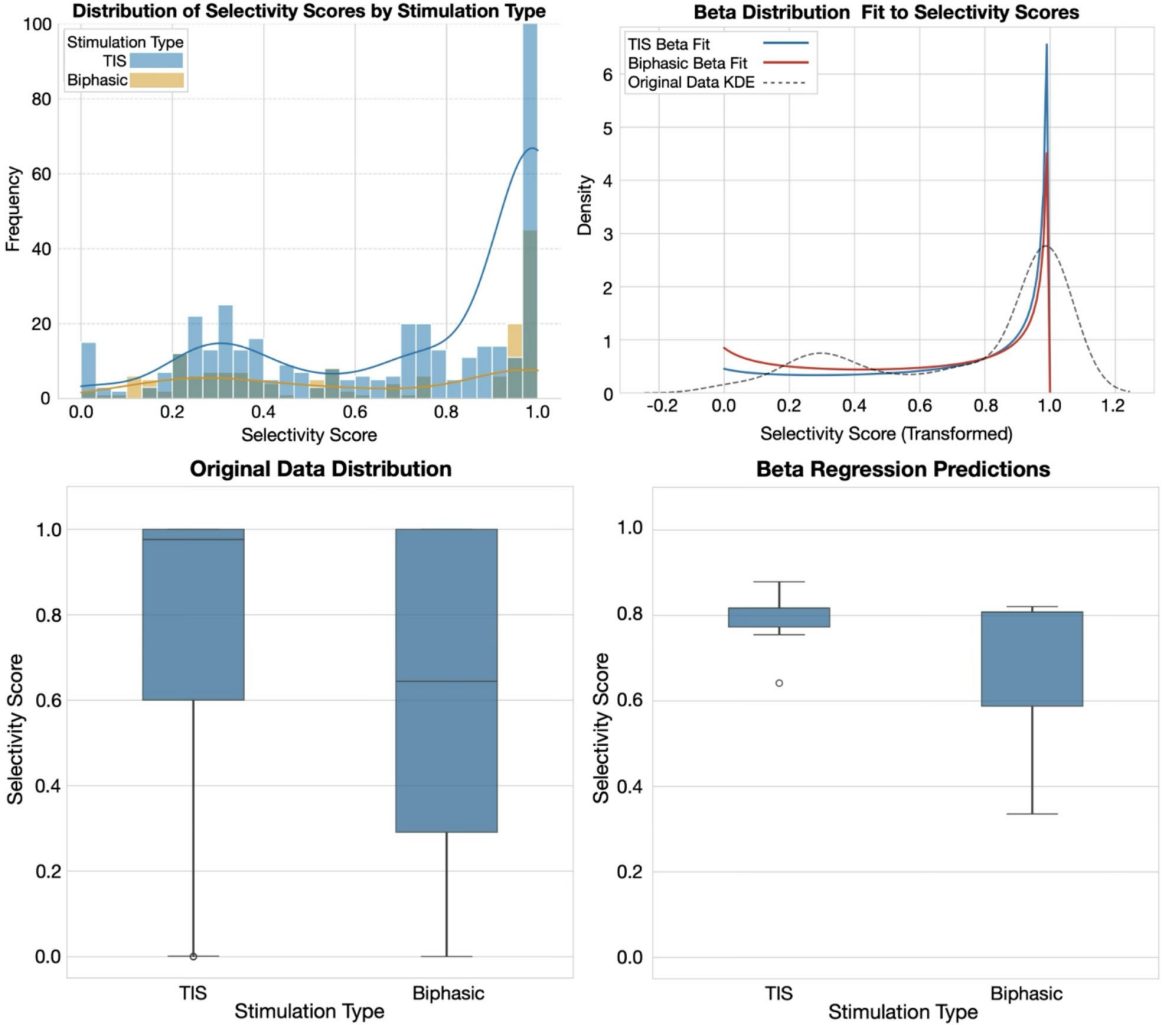
Beta regression modeling of stimulation type and their predicted selectivity scores.

Since the selectivity scores are proportional and bounded by [0,1], we then used the beta regression model [40] to determine the effect of biphasic and TIS stimulation which were coded as categorical predictors (Fig. 11). After transforming the data using a logit-link function, the beta model showed a significant positive effect of TIS on selectivity (β = 2.75, *p* < 0.005). This finding was further supported by non-parametric analysis, with the Mann-Whitney U test on the original scores indicating a significant difference between stimulation types (*p* < 0.001). The magnitude of this effect was moderate, as evidenced by Cohen’s d (0.512) and Cliff’s Delta (0.270). Together, these complementary statistical approaches provide strong evidence that TIS yields higher selectivity than biphasic stimulation. Therefore, our analysis revealed a consistent positive effect of TIS compared to biphasic stimulation on selectivity scores.

Taken together, these findings highlight the effectiveness of our semi-supervised analysis pipeline in revealing the superior selectivity and movement variety elicited by extraneural temporal interference stimulation compared to standard biphasic stimulation.

## Discussion

Temporal interference stimulation (TIS) has emerged as a promising non-invasive technique for selectively targeting regions of the brain, showing potential clinical effects [41, 42]. However, resolving debates around the mechanisms underlying TIS requires detailed quantification of the stimulation results. Thus, we leverage advances in computer vision and data science to measure and contrast the movement patterns achieved using multiple stimulation types and configurations.

One of the proposed advantages of TIS is its ability to penetrate deeper tissues without functionally activating overlaying structures. Mechanistically, Mirzakhalili et al. demonstrated in silico that this phenomena may be due to neurons acting as demodulators of the high frequency signal. The model showed a “sandwich” pattern of activation [23], where regions directly on the electrodes are blocked or attenuated and those in the middle of the electrodes are active with phasic firing based on the low frequency envelope. However, some regions close to the electrode can exhibit tonic firing, as the model is dependent on network effects and ion channel nonlinearities [43–45].

On the other hand, peripheral nerve studies indicate a different biophysical mechanism where a simple linear integration model was demonstrated, and amplitude-modulated (AM) kHz stimulation was sufficient to induce movement [25, 26]. Despite the different proposed mechanisms, it was still demonstrated that muscle selectivity was achieved using TIS in the sciatic nerve [25]. This was corroborated in our experiments, showing that TIS led to more selective movements compared to traditional biphasic stimulation. Notably, applying two electrical sine waves with similar non-AM high frequencies (2 kHz) induced an initial jerk reaction but immediately led to limb relaxation, possibly due to the high frequency attenuation or conduction block. Whereas, having a delta frequency or low frequency envelope resulted in a consistent phasic movement of the hindlimb.

These observations suggest that TIS can achieve selective tissue activation through the combined effects of high frequency conduction block and low frequency stimulation that leads to spatial localization. Our behavioral analyses suggest that TIS produced more specific and diverse movements compared to biphasic stimulation that lack a carrier frequency or pulse width modulation. This raises the question of whether other arbitrary waveforms with amplitude or pulse modulation could yield similar movement selectivity, since comparable stimulation efficiencies were demonstrated in the brain and nerve [26, 46].

Furthermore, TIS has been shown to require lower amplitudes than transcutaneous electrical neurostimulation (TENS) to activate peripheral nerves like the sciatic and hypoglossal nerves [27, 30]. However, compared to transcranial alternating current stimulation (tACS), TIS necessitates higher currents to modulate deep brain regions and may be better suited for disrupting synchronization and spike timing [43]. Thus, finding an optimal location and application for TIS remains important. Nonetheless, the potential of TIS for localized focal stimulation can be leveraged and further enhanced through multipolar arrangements [47] or current steering modalities [25, 42].

The selectivity of TIS is also influenced by the arrangement of the electrode configurations used for stimulation. Research indicates that multi-contact electrodes can enhance the selectivity of peripheral nerve stimulation by allowing for independent activation of different populations of nerve fibers within a single nerve [34, 48]. This is particularly relevant in the context of TIS, where the configuration of electrodes can be optimized to achieve the desired stimulation patterns while minimizing invasiveness [47, 49]. We tested 165 TIS configurations and 151 biphasic configurations using both manual and automated stimulations across multiple trials and found that overall, TIS configurations were more selective. Even if we increased the number of electrodes for biphasic stimulation, from bipolar to tripolar, TIS still performed better, and interestingly, bipolar biphasic stimulation selectivity trended slightly higher than tripolar biphasic cofigurations (Supplementary Fig. 6). Thus, a simple increase in active electrode count may not account for increased selectivity. Nonetheless, a recommendation would be to test selectivity as a function of active channel number (≥ 4 electrodes) in both biphasic and TIS. Additionally, outcomes varied depending on the subject and specific trial, likely due to inter-subject anatomical differences, varying contact impedances, and minor electrode displacement during movement. Addressing these variables through personalized anatomical imaging and impedance-based modeling could provide better insights for clinical applications [50, 51].

Aside from addressing inter-subject and trial variability, an extension of the study would be to determine whether the complex and diverse movements induced by TIS can be combined in a sequentially desired motion that is more functional than simple muscle activations of conventional stimulation techniques. Given the specific mapping of electrode configuration to movement cluster/syllable in TIS, this may be useful for motions like walking or reaching which require specific muscle groups that can be selectively activated by different fascicles with the right timing and electrode configuration. By testing more parameters across frequencies, amplitudes, and waveforms; and searching through all the possible induced movements by TIS, an adaptive closed-loop algorithm can be designed to try and generate a desired motion [52, 53]. Additionally, using machine learning to uncover more complex patterns and combinations of TIS waveforms and configurations would enhance the applicability of TIS for neurorehabilitation.

Therefore, our results demonstrate the potential of TIS in peripheral nerves to selectively induce multiple motions in the limb, which illustrates an advantage over standard biphasic stimulation. As this is the first study to demonstrate a wide variety of selective movements in TIS, the behavioral clusters and syllables extracted from this research can be used as building blocks for constructing more functional motion such as walking and grasping. Given that spinal cord stimulation using biphasic pulses has been shown to restore walking in patients with spinal cord injuries [54, 55], a more localized and selective approach like peripheral nerve TIS presents an exciting complement to the existing toolbox of neuromodulation strategies.

## Conclusion

Through a combination of computer vision and unsupervised learning techniques, we quantified the results of multiple peripheral nerve stimulation strategies. We developed a 64-channel extraneural cuff electrode connected to stimulation modules capable of temporal interference stimulation (TIS), which was evaluated for its potential to elicit selective limb movements. Our results suggest that TIS produced more selective and diverse hindlimb movements compared to conventional biphasic stimulation. Integrating TIS with extraneural cuff electrodes provided a less invasive approach for targeted peripheral nerve activation compared to other fascicle-selective methods such as intraneural and regenerative interfaces. This offers safety and functional advantages over existing neural interfaces and stimulation modalities. The ability of TIS to selectively target deep nerve activity, when combined with optimized electrode designs, positions extraneural TIS as a useful tool for both clinical and research applications. Furthermore, by incorporating automated and personalized physiological assessments, such as imaging for determining the subject’s nerve anatomy, this interface has the potential to enhance the selectivity and efficacy of peripheral nerve therapies. One such example demonstrated here is for movement rehabilitation in individuals with spinal cord injury, where selective nerve stimulation and movement induction can ultimately improve motor function and quality of life.

## Supplementary Information

Below is the link to the electronic supplementary material.

Supplementary Material 1.

## Data Availability

Data and code that support the findings of this study have been uploaded to: https://github.com/josh-oloro/tis-selectivity and videos can be made available upon request.
