# Supplementary material for: Temporal interference stimulation of peripheral nerves induces functionally diverse limb movements revealed by automated pose estimation and unsupervised behavioral analysis
Source: J Neuroeng Rehabil. 2025 Dec 29;23:29. doi: 10.1186/s12984-025-01825-3 (PMC12825211; doi:10.1186/s12984-025-01825-3)
Supplement: Supplementary file 1 — Supplementary Material 1. [file 12984_2025_1825_MOESM1_ESM.docx]

| **Biphasic Stimulation** | | | | **Temporal Interference Stimulation** | | | |
| --- | --- | --- | --- | --- | --- | --- | --- |
| *Animal ID* | *Number of Locations* | *Type of Arrangement* | *Number of Stimulation Trials* | *Animal ID* | *Number of Locations* | *Type of Arrangement* | *Number of Stimulation Trials* |
| **Animal A** | 1 | 2-ch | 4 | **Animal B** | 1 | 4-ch | 4 |
| **Animal C** | 2 | 2-ch | 16 *(4 per location)* | **Animal D** | 3 | 4-ch | 9 *(3 per location)* |
| **Animal F** | 2 | 3-ch longitudinal | 12 *(6 per location)* | **Animal F** | 2 | 4-ch | 12 *(6 per location)* |
| **Animal H** | 35 | 2-ch | 35 | **Animal G** | 31 | 4-ch | 31 |
|  | 3 | 3-ch longitudinal | 3 |  |  |  |  |
|  | 3 | 3-ch transverse | 3 |  |  |  |  |
| **Animal K** | 32 | 2-ch | 64 *(2 per location)* | **Animal I** | 128 | 4-ch | 384 *(3 per location)* |
| **Animal L** | 25 | 2-ch | 50 *(2 per location)* | **Animal J** | 128 *(same as previous animal)* | 4-ch | 256 *(2 per location)* |
|  | 16 | 2-ch | 16 | **Animal L** | 128 *(same as previous animal)* | 4-ch | 256 *(2 per location)* |
|  | 16 | 3-ch longitudinal | 16 |  |  |  |  |
|  | 16 | 3-ch transverse | 16 |  |  |  |  |
|  |  |  |  |  |  |  |  |
| **Total configurations tested** | **151** |  |  | **Total configurations tested** | **165** |  |  |
|  |  |  |  |  |  |  |  |

**Supplementary Figure 1.** Table of experimental configurations tested across different animals. The following electrode arrangements were tested: Temporal Interference Stimulation (TIS) – 4-channel, Biphasic Stimulation – 2-channel (BP), 3-channel longitudinal (TL), 3-channel transverse (TT). These electrode arrangements were activated across different locations on the nerve cuff resulting in 151 unique configurations for biphasic stimulation and 165 configurations for TIS.


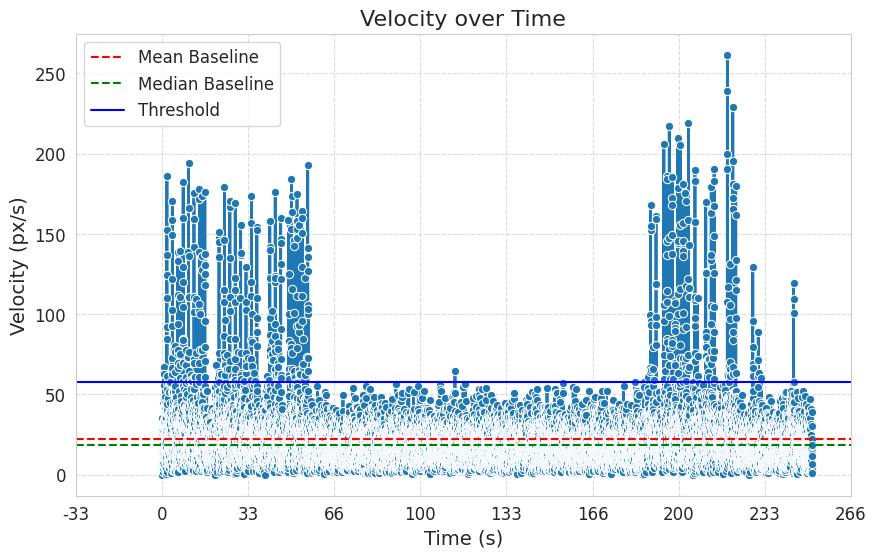


**Supplementary Figure 2.** Sample limb velocity over time with a threshold to remove consecutive time points with no movement.


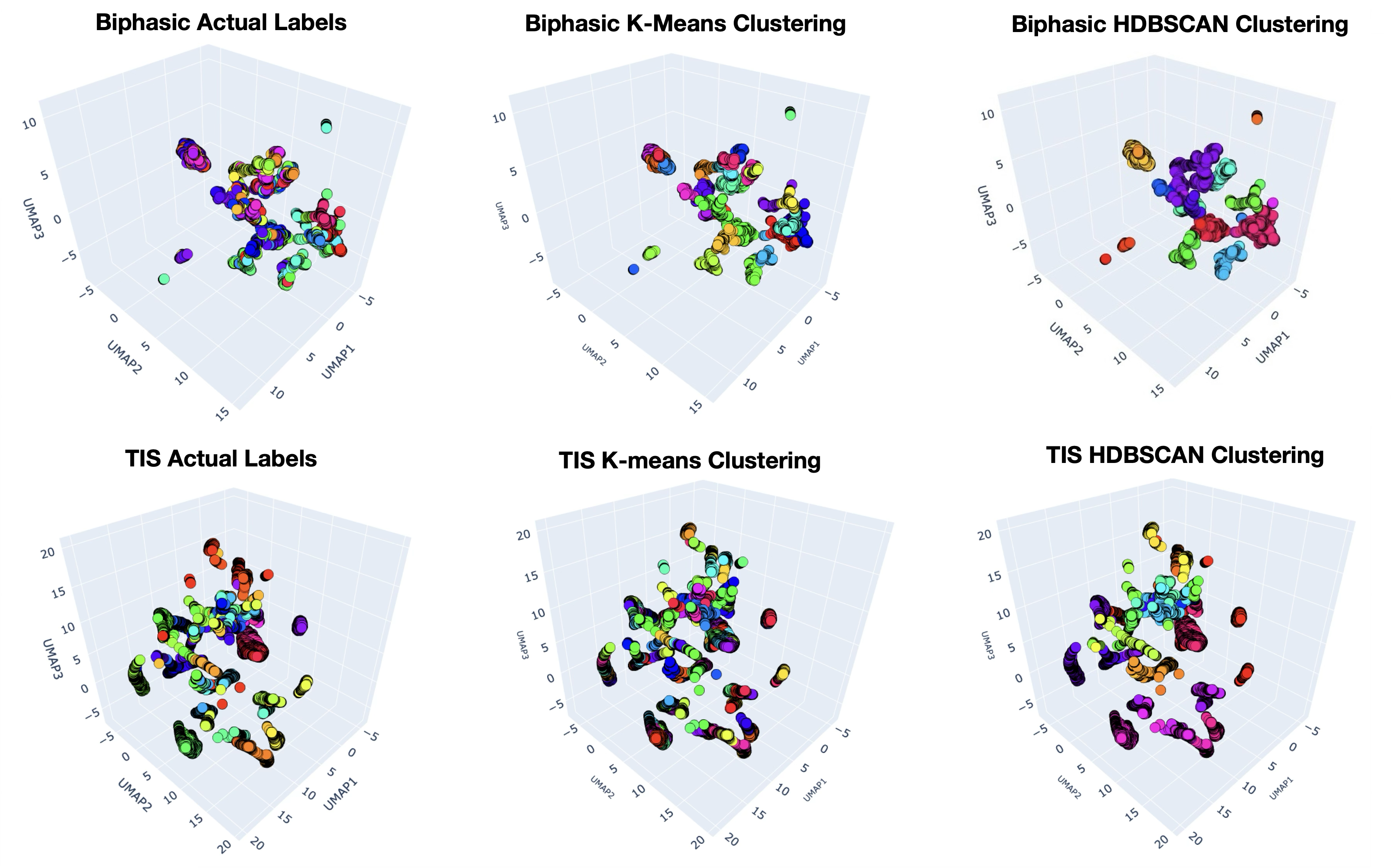


**Supplementary Figure 3.** Side-to-side comparison of the electrode configuration labels (actual labels), K-means clustering labels, and HDBSCAN clustering labels from left to right. TIS showed more consistent clustering that follow the actual electrode configuration labels when using HDBSCAN clustering as opposed to K-means clustering.

| Modality | UMAP parameters | HDBSCAN parameters | Silhouette | ARI | AMI | Clusters |
| --- | --- | --- | --- | --- | --- | --- |
| Biphasic (Optimized) | n_neighbors=50,  min_dist=0 | min_cluster_size=30, min_samples=5 | **0.512** | 0.059 | **0.332** | 6 |
| Biphasic (Shared UMAP only) | n_neighbors=113,  min_dist=0 | min_cluster_size=30, min_samples=5 | 0.331 | 0.004 | 0.111 | 5 |
| Biphasic (Fully Shared) | n_neighbors=113,  min_dist=0 | min_cluster_size=79, min_samples=152 | 0.508 | 0.036 | 0.252 | 5 |
| Biphasic (Shuffled) | n_neighbors=50,  min_dist=0 | min_cluster_size=30, min_samples=5 | 0.035 | 0.000 | 0.000 | 2 |
| Biphasic (Gaussian Noise) | n_neighbors=50,  min_dist=0 | min_cluster_size=30, min_samples=5 | 0.224 | 0.005 | 0.142 | 8 |
| TIS (Optimized) | n_neighbors=177,  min_dist=0 | min_cluster_size=128, min_samples=300 | **0.744** | 0.153 | **0.686** | 24 |
| TIS (Shared UMAP only) | n_neighbors=113,  min_dist=0 | min_cluster_size=128, min_samples=300 | 0.700 | 0.158 | 0.693 | 27 |
| TIS (Fully Shared) | n_neighbors=113,  min_dist=0 | min_cluster_size=79, min_samples=152 | 0.608 | 0.170 | 0.699 | 27 |
| TIS (Shuffled) | n_neighbors=177,  min_dist=0 | min_cluster_size=128, min_samples=300 | 0.644 | 0.000 | 0.000 | 21 |
| TIS (Gaussian Noise) | n_neighbors=177,  min_dist=0 | min_cluster_size=128, min_samples=300 | 0.585 | 0.015 | 0.309 | 6 |

**Supplementary Figure 4.** Clustering metrics of optimized or shared UMAP-HDBSCAN parameters and surrogate noise data for control analysis. Hyperparameters for UMAP and HDBSCAN were optimized for each dataset (TIS and Biphasic) separately using Bayesian optimization to attain the best silhouette score. Additionally, control analyses were done where (i) UMAP parameters were similar; (ii) both UMAP and HDBSCAN parameters were similar (“fully shared”); (iii) the position vectors were randomly shuffled across time (row-wise); and (iv) Gaussian noise was added in TIS and Biphasic datasets. TIS consistently showed higher AMI and ARI scores than Biphasic stimulation across different hyperparameters, suggesting better movement selectivity. Whereas, shuffling the data or adding Gaussian noise removed these differences, suggesting that the original data distribution had meaningful structures which differed from chance.

**
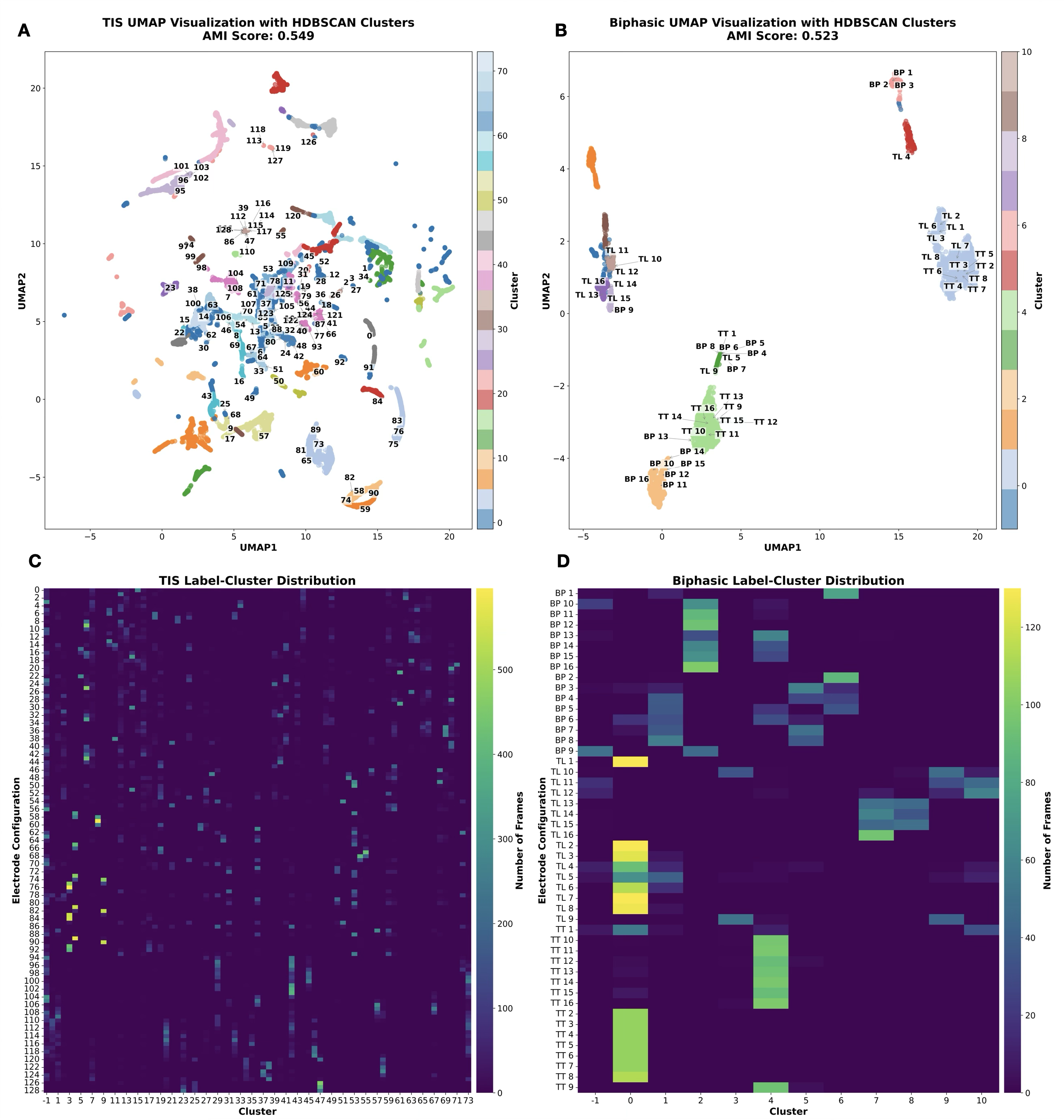
**

**Supplementary Figure 5.** Clustering analysis of TIS and Biphasic stimulation in the same rat with various channel number configurations. BP – bipolar (2-ch) configurations, TL – tripolar (3-ch) longitudinal configurations, and TT – tripolar transverse configurations for biphasic stimulation. TIS (4-ch) displayed a higher AMI score and more varied cluster distribution.


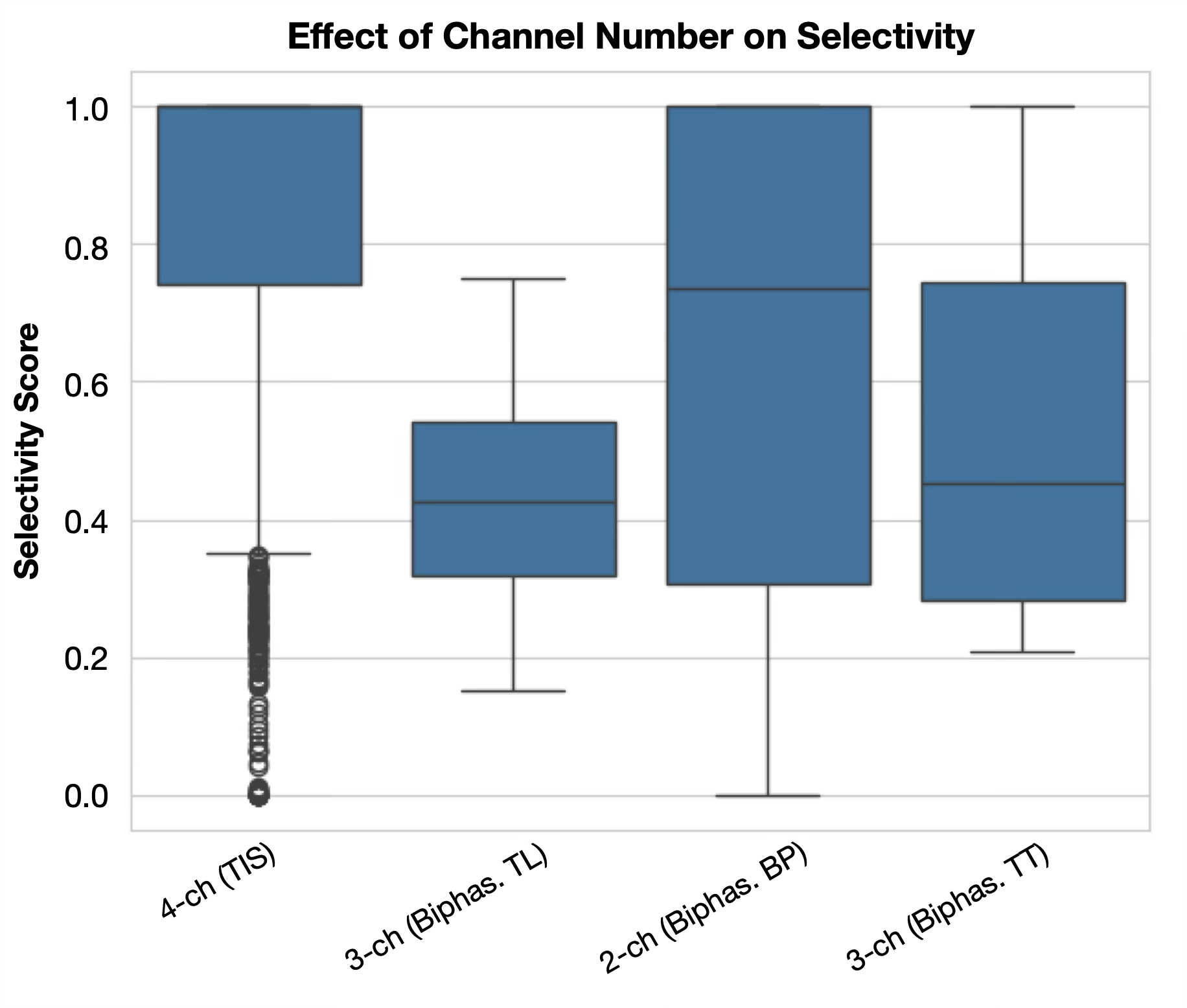


**Supplementary Figure 6.** Effect of Channel Number on Selectivity Score. The selectivity score is measured using entropy calculations discussed in the main text, and these were grouped according to number of channels (ch) used. Temporal interference stimulation (TIS) with 4 channels showed the highest distribution of selectivity scores; however, 3-channel biphasic stimulation (TL – tripolar longitudinal, TT – tripolar transverse) had lower selectivity scores while 2-channel (BP – bipolar) biphasic stimulation had a high variance.
